# Clostridium septicum sepsis and colorectal cancer - a reminder

**DOI:** 10.1186/1477-7819-7-73

**Published:** 2009-10-06

**Authors:** Nazzia N Mirza, Jonathon M McCloud, Mark J Cheetham

**Affiliations:** 1 Department of Surgery, Royal Shrewsbury Hospital, Mytton Oak Road, Shrewsbury, UK

## Abstract

**Background:**

Spontaneous clostridium septicum infections are rare and are associated with a high mortality. Association of clostridium infection with colorectal malignancies have been previously reported and most cases are described in tumours of the ascending colon. We report our experience of clostridium septicum infection in the presence of tumour perforation in a series of two patients as a reminder of its association with sepsis in the presence of colorectal malignancy.

**Case Presentation:**

We isolated clostridium septicum infection in a series of two patients admitted as emergencies. One patient was found to have a perforated caecal tumour intraoperatively whilst the other had a perforated rectal tumour. The clinical outcome and management of each case are reported and underlying reasons for variations in outcome are discussed.

**Conclusion:**

Although uncomman, the possibility of clostridium septicum sepsis should be borne in mind in patients who present with underlying malignancy and have sepsis. The cumulative effect of sepsis and malignant perforation is associated with a high morbidity and mortality. Awareness and early diagnosis of clostridium septicum may improve the prognosis of what is usually regarded as a fatal infection.

## Background

Spontaneous Clostridium septicum infections are rare and are associated with cyclical neutropenia, diabetes mellitus and immunosupression [1-3]. In addition, a strong association of spontaneous C.septicum infection with haematological and colorectal malignancies has been reported [1-6].

C.septicum sepsis is often fulminant with reported mortality rates of approximately 60% [1]. Most cases of C.septicum reported in colonic malignancies are described in tumours of the ascending colon [5,6]. We isolated C.septicum infection in the presence of tumour perforation in a series of two patients. The cumulative effect of sepsis and malignant perforation is associated with significant morbidity and mortality [7,8]. Here we report our experience of C.septicum infection as a reminder of its association with sepsis in the presence of colorectal malignancy and discuss the clinical outcomes.

## Case presentation

### Case 1

**Figure 1:**
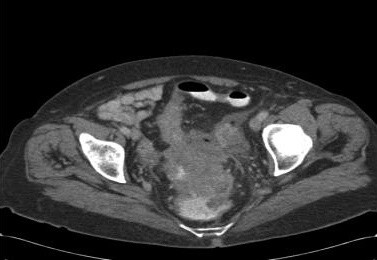
**CT scan demonstrating a rectosigmoid mass with local perforation**.

A 69-year-old woman presented with a 6-month history of diarrhoea and left iliac fossa pain of short duration that had progressively worsened prompting emergency admission. Since the onset of her symptoms, she had not sought any medical advice and had no records of previous hospital admissions with the above symptoms. Her only past medical history was of peptic ulcer disease. On examination, she had a temperature of 37.8°C and was tender in the lower abdomen but had no signs of peritonitis. No other clinical signs of septicaemia were present. Initial investigations revealed a raised white cell count of 23.0 × 10^9 ^(neutrophilia) and an elevated CRP. Urine microscopy revealed white cells and culture confirmed an E. coli urinary tract infection. She was treated initially with intravenous gentamicin, benzyl penicillin and metronidazole for an intra abdominal cause of sepsis according to the hospital microbiology protocol. The following day, she developed swinging pyrexia and blood cultures were taken. She proceeded to have a CT scan that revealed a distal sigmoid mass with extensive circumferential wall thickening and air beyond the bowel wall lumen suggesting localised perforation. There was no evidence of a pneumoperitoneum or of metastatic disease (Figure 1). In view of the clinical and radiological findings, an emergency laparotomy was performed on day 3 of admission. Immediately pre-operatively, she was found to have C.septicum positive blood cultures. Preoperatively, 1.2 g of benzylpenicillin and 500 mg of metronidazole were administered intravenously and gentamicin was discontinued following microbiology advice. Intra operatively, a bulky inflammatory mass was identified in the upper rectum with localised perforation and pus. In view of the sepsis and oedematous rectal wall, a low Hartmann's procedure was carried out rather than a restorative resection. Cultures from the pus swab taken intra-operatively subsequently grew a heavy growth of C.septicum. Postoperatively she remained on intravenous antibiotics (benzylpenicillin 1.2 g QDS, and metronidazole 500 mg tds) for 5 days and made an uneventful recovery.

Histology revealed a poorly differentiated Dukes B (pT3N0 Mx) adenocarcinoma. In view of the fact that she had a perforated rectal cancer, she was subsequently referred for adjuvant chemotherapy.

### Case 2

A 80 -year -old woman was admitted as an emergency with a six-week history of progressively worsening right upper quadrant pain associated with nausea, vomiting and anorexia. She had not noticed any change in her bowel habit but had been using laxatives regularly.

She had significant co-morbidities including Type II Diabetes mellitus, chronic renal failure for which she required erythropoetin injections, polymyalgia rheumatica, hypertension, ischaemic heart disease, deep vein thrombosis and gout. She had previously undergone a cholecystectomy and a total abdominal hysterectomy and bilateral oophorectomy.

On examination, she had a temperature of 37.2°C and was tender in the RUQ. Initial investigations revealed a profound anaemia with haemoglobin of 5 g/l (hypochromic microcytic picture), an elevated white cell count and renal impairment. She had an isolated raised alkaline phosphatase and all of the other liver function tests were within normal parameters. An ultrasound scan of the abdomen revealed a possible ascending colon mass and she proceeded to have a CT scan to further evaluate these findings. CT confirmed an ascending colon mass with suspicious lesions in the liver suggestive of possible metastases.

She underwent an emergency right hemicolectomy day 9 of admission. (Preoperatively a single dose of Gentamicin (7 mg/kg), benzylpenicillin 1.2 g and metronidazole 500 mg were administered. Intraoperatively, a perforated retroperitoneal tethered ascending colon tumour was found.

In view of her significant underlying co-morbidities, she was transferred to the high dependency unit for postoperative care.

Day 3 of her post-operative course was complicated by acidosis, worsening renal function, abdominal distention and worsening abdominal pain. She also developed a wound infection that became apparent the same day. An urgent CT scan without contrast was carried out which showed free fluid and pockets of air with in the fluid. No definite evidence of anastomotic leak was identified. In view of her deteriorating condition, an emergency laparotomy was performed. This was carried out 14 days later following her previous surgery. Areas of necrosis and pus were identified in the abdominal wall, which was debrided and tissue was sent for culture. Approximately 4 L of straw-coloured fluid was drained which was in keeping with ascites. There was no evidence of pus or anastomotic leak. A thorough washout was carried out and drains were placed. Tissue cultures of the abdominal wall showed a heavy growth of Coliform organisms and a moderate growth of positive for C.septicum and Faecal streptococcus. The tumour was noted to have perforated intraoperatively and these findings were also confirmed on histology and is likely to have been the origin of the abdominal wound infection. In view of this, vancomycin and metronidazole were used to treat sepsis. Her condition remained critical and she died of overwhelming sepsis 2 days later.

Histology subsequently confirmed a Dukes C1 (pT4N1 Mx) mucinous adenocarcinoma of the ascending colon.

## Discussion

Clostridium septicum is an anaerobic gram-positive spore forming bacillus and is a causative agent of gas gangrene or atraumatic myonecrosis [9]. Production of the alpha toxin by this pathogen is an essential virulence factor [1]. C.Septicum infections are known to co exist with malignancy and it is thought that disruption of the normal mucosal barrier caused by mucosal ulceration of the tumour surface and haematogenous invasion allow a portal of entry for the bacteria [6]. One explanation as to why clostridium infections are present in malignancy is that anaerobic glycolysis of the tumour provides a hypoxic and acidic environment that may be conducive to spore germination thus leading to infection [3]. As previously mentioned C.septicum infections are rare. In one study 17 cases of C.septicum infection were reported over a 12- year period of which associated colorectal malignancy was identified in 4 cases [10]. A further study reported 5 cases of C.septicum infection over a 10-year study period of which 2 of the 5 patients had associated colorectal malignancy [3]. Clinical findings of localised pain, inflammation, crepitation and generalised toxicity are highly suggestive of atraumatic myonecrosis [11].

Although C.septicum infection is an uncommon diagnosis, early identification, administration of appropriate antibiotics and surgery in which the sepsis was "resectable" improved outcome in case 1. In case 2 the patient had extensive abdominal wall necrosis and retroperitoneal sepsis that was not resectable and delay in diagnosis along with patient's significant co-morbidities contributed towards her poor prognosis.

The possibility of C.septicum infection should be borne in mind in patients who present with an underlying malignancy and have signs of sepsis. Timely diagnosis is of the utmost importance for proper treatment. Cultures should be obtained to confirm the diagnosis and aid in future antibiotic regimens. Data from previous studies have shown that C.septicum is susceptible to a wide range of antibiotics such as clindamycin, penicillin and metronidazole [4]. Although hyperbaric oxygen therapy is thought of as a useful adjunct [12], no adequately controlled trials of hyperbaric oxygen treatment exist [13-15]. In addition, C septicum may not be as responsive to hyperbaric oxygen as other clostridium species [16]. Antibiotics and surgical treatment remains the mainstay of treatment. Awareness and early diagnosis of C.septicum may improve the prognosis of what is regarded as a fatal infection.

## Consent

Written informed consent was obtained from the patient for publication of this case report and accompanying images. A copy of the written consent is available for review by the Editor-in-Chief of this journal

## Competing interests

The authors declare that they have no competing interests.

## Authors' contributions

MJC contributed to the study design, manuscript prerparation, editing and has approved the final version. JMM contributed to manuscript preparation. NNM was involved in the manuscript preparation and final editing. All authors approved the final manuscript.
